# Chasing genes at high‐altitude

**DOI:** 10.1113/EP091877

**Published:** 2024-09-19

**Authors:** Samantha Sharma, Qadar Pasha

**Affiliations:** ^1^ Department of Medical and Molecular Genetics Indiana University Indianapolis Indiana USA; ^2^ Institute of Hypoxia Research New Delhi India

Extreme environments impose stringent selective pressures for life on Earth that have undoubtedly led to organisms with specialized adaptations to survive. These physiological adaptations represent a frontier in biological research, offering unique opportunities to elucidate the fundamental mechanisms underlying survival and homeostasis (Rappaport & Oliverio, 2023). Through genomic studies and molecular analyses, investigators have unraveled intricate mechanisms of natural selection that govern the response to environmental stressors and offer a glimpse into the evolutionary journey of the diverse life forms on Earth (Ando et al., 2021; Goh et al., 2023; Mishra, Kohli, et al., 2015). One such instance is life at high altitudes (>2400 m), whereby diverse human populations and numerous animal species have defied the odds of survival by successfully inhabiting the landscapes of the Himalayan/Ladakh/Qinghai‐Tibetan Plateaus, the Andean Altiplano and the Ethiopian Semien Plateau over evolutionary time scales (Mishra, Mohammad, et al., 2015; West et al., 2012; Witt & Huerta‐Sanchez, 2019).

In this pursuit of elucidating the genetic basis of high‐altitude adaptation, our recent study utilized a comprehensive (>1 million single nucleotide polymorphisms (SNPs)) genome‐wide analysis on a diverse cohort encompassing one low‐altitude (<500 m) and three native high‐altitude populations inhabiting at varying altitudes of the Himalayan region (>2400 m): Nubra valley (NU) at 3048 m, Sakti (SKT) at 3812 m and Tso Moriri villages (TK) at 4522 m (Sharma et al., 2024). We revealed a clear correlation between increasing altitude and genetic variability and associated physiological traits, and identified 86 SNPs as pivotal players in high‐altitude adaptation with frequency mapping, further revealing 38 putatively adaptive alleles and specific haplotypes (Figure 1). A substantial number of the SNPs identified were found on chromosomes 1 and 2, particularly concentrated in genes *EGLN1* (*rs973253*, *rs2486736*, *rs480902*, *rs2486729*, and *rs2808611*); *EPAS1* (*rs2121266*, *rs4953353*, and *rs7571218*) and *SPRTN* (*rs1009227* and *rs2749717*), all of which have been previously implicated in the physiological response to high‐altitude environments. (Huerta‐Sanchez et al., 2013; Mishra et al., 2013; Peng et al., 2017).

**FIGURE 1:**
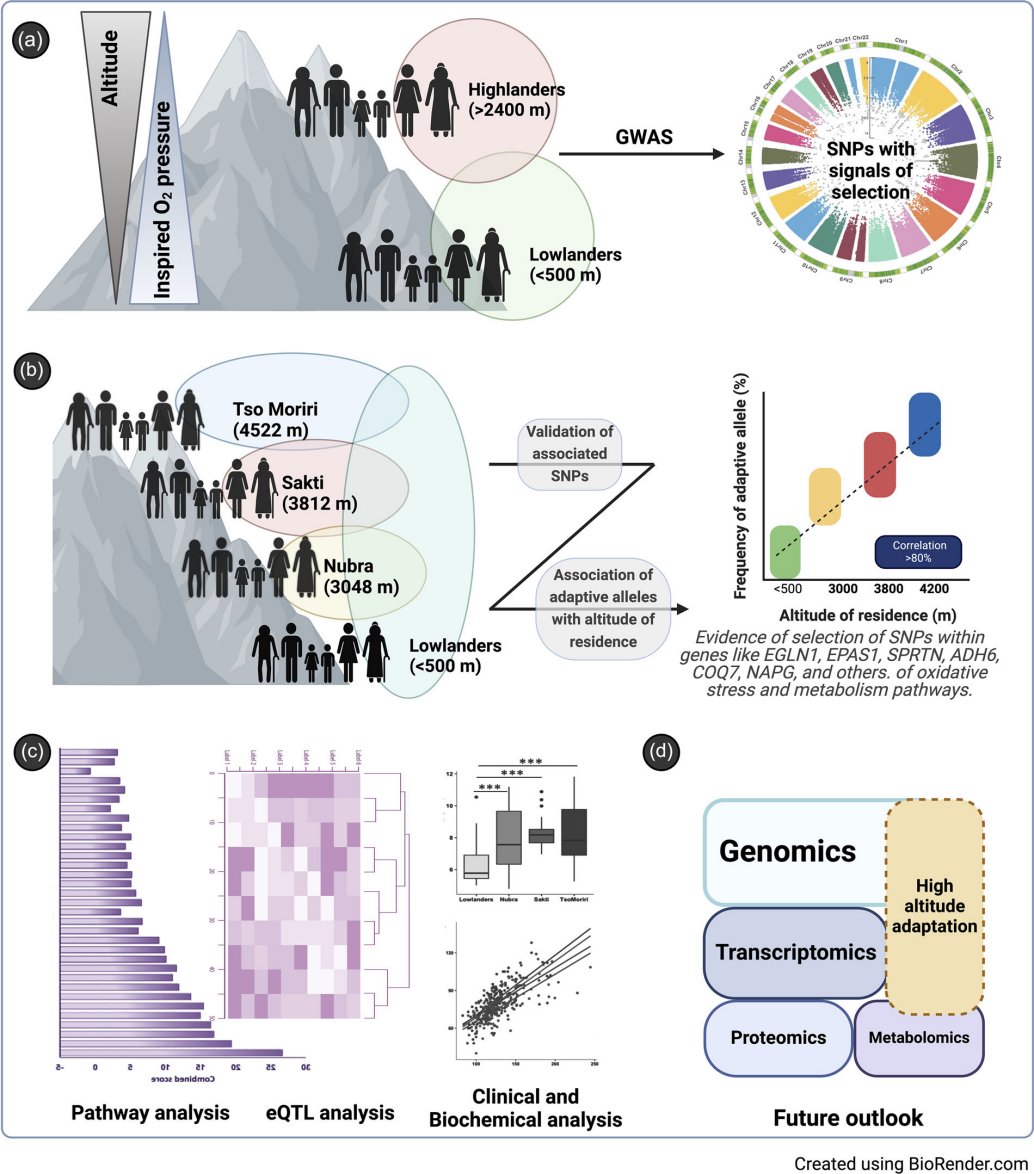
Genotype–phenotype mechanisms in high‐altitude adaptation. (a) Genome‐wide association study (GWAS) comparing the native high‐altitude (highlanders; >2400 m) and low‐altitude (lowlanders; <500 m) populations identify SNPs linked to high‐altitude adaptation. (b) Validation of SNPs in a larger cohort of high‐altitude natives specifically from Nubra valley (3048 m), Sakti (SKT) (3812 m) and Tso Moriri villages (4522 m), along with the lowlanders. Evidence of selection of adaptive SNPs within genes like *EGLN1*, *EPAS1*, *SPRTN*, *ADH6*, *COQ7*, *NAPG* and others. (c) Pathway enrichment analysis using KEGG pathways followed by eQTL analysis confirms the functional SNPs. Clinical and biochemical data supported altitude‐dependent trends, with adaptive alleles correlating significantly with these parameters. (d) Comprehensive studies integrating genomics, transcriptomics, proteomics and metabolomics are essential for a deeper understanding of high‐altitude adaptation in worldwide populations.

Genetic markers are instrumental in shaping the adaptive phenotypes observed in native populations residing at high altitudes (Bigham, 2016). In humans, the *EPAS1* gene is a master regulator of the hypoxia‐inducible factor (HIF) pathway, and has emerged as a central player in high‐altitude adaptation. Variants in *EPAS1* are associated with a blunted erythropoietic response and reduced haemoglobin concentration in Tibetan and Andean highlanders, which is suggested to play a protective role against chronic mountain sickness (Beall et al., 2010; Lawrence et al., 2024; Peng et al., 2017; Yi et al., 2010). Similarly, genetic variations in *EGLN1* have been linked to improved oxygen utilization and energy metabolism at high altitude (Bigham et al., 2010; Brutsaert et al., 2019; Ge et al., 2015; Mishra et al., 2013; Simonson et al., 2010; Wang et al., 2011). Notably, putatively adaptive alleles of genes such as *ADH6*, *COQ7*, and *NAPG* were linked to decreased systolic blood pressure, while variants in *SPRTN* and *EGLN1* correlated with elevated levels of 8‐isoPGF2α, a marker of oxidative stress, further emphasizing the intricate relationship between genetic adaptations and physiological responses to high‐altitude environments (Sharma et al., 2024).

Recent research has also indicated that gradual exposure to increasing altitudes can influence the epigenome, particularly the *EPAS1* gene, capable of altering successful acclimatization to high altitude (Childebayeva et al., 2019). In support, we identified genes that were exclusively associated with the TK population. These included genes involved in the oxidative stress response pathways such as *DUOXA1*, *CHST11*, and *SMOX* (Sharma et al., 2024), which have been previously implicated in the adaptation and maladaptation to hypoxia at high altitude (Bigham et al., 2009; Gaur et al., 2020; Zhou et al., 2013).

Understanding the genetic and epigenetic basis of high‐altitude adaptation holds significant translational potential for medicine. Insights into the molecular mechanisms underlying hypoxia tolerance could inform the development of novel therapies for conditions characterized by arterial hypoxaemia, such as chronic obstructive pulmonary disease, acute lung injury, and sleep apnoea. Moreover, investigating the interplay between genetic, epigenetic, and physiological adaptations could lead to personalized approaches for enhancing human performance in extreme environments, such as high‐altitude mountaineering. Continued research in high‐altitude genomics, physiology, and epigenetics will not only deepen our understanding of evolutionary processes but also pave the way for innovative interventions to improve human health and resilience in challenging environments.

## AUTHOR CONTRIBUTIONS

Conception or design of the work: Samantha Sharma and Qadar Pasha. Drafting of the work or revising it critically for important intellectual content: Samantha Sharma and Qadar Pasha. Both authors have read and approved the final version of this manuscript and agree to be accountable for all aspects of the work in ensuring that questions related to the accuracy or integrity of any part of the work are appropriately investigated and resolved. All persons designated as authors qualify for authorship, and all those who qualify for authorship are listed.

## CONFLICT OF INTEREST

None declared.

## FUNDING INFORMATION

None.
